# Skin Sparing Mastectomy and Immediate Breast Reconstruction (SSMIR) for early breast cancer: Eight years single institution experience

**DOI:** 10.1186/1477-7819-6-43

**Published:** 2008-04-27

**Authors:** Ramesh Omranipour, Jean yves Bobin, Mustafa Esouyeh

**Affiliations:** 1 Current-Department of Surgical Oncology, Cancer Institute, Tehran University Of Medical Science, Tehran, Iran; 2 Department of Surgical Oncology, Lyon Sud Hospital, 69495 Pierre Benite cedex, France

## Abstract

**Background:**

**S**kin Sparing Mastectomy (SSM) and immediate breast reconstruction has become increasingly popular as an effective treatment for patients with breast carcinoma. The aim of this study was to evaluate the clinical outcome of skin sparing mastectomy in early breast cancer at a single population-based institution.

**Methods:**

Records of ninety-five consecutive patients with operable breast cancer who had skin-sparing mastectomy and immediate breast reconstructions between 1995 and 2003 were reviewed. Patient and tumor characteristic, type of reconstruction, postoperative complications, aesthetic results and incidence of recurrence were analyzed.

**Results:**

Mean age of the patients was 51.6(range 33–72) years. The AJCC pathologic stages were 0 (n = 51, 53.7%), I (n = 20, 21.1%), and II (n = 2, 2.1%). Twenty of the patients had recurrent disease (21.1%). The immediate breast reconstructions were performed with autologus tissue including *latissimus dorsi *musculocutaneous flap in 63 (66.3%) patients and *transverse rectus abdominis *myocutaneous (TRAM) flap in 4 (4.2%) patients. Implants were used in 28 (29.4%) patients. The average hospital stay was 7.7 days. Flap complication occurred in seven (10.4%) patients resulting in four (6%) re-operations and there were no delay in accomplishing postoperative adjuvant therapy. At a median follow-up of 69 months (range 48 to 144), local recurrence was seen in one patient (1.1%) and systemic recurrence was seen in two patients (2.1%).

**Conclusion:**

Skin sparing mastectomy and immediate breast reconstruction for early breast cancer is associated with low morbidity and low rate of local recurrence.

## Background

Skin sparing mastectomy (SSM) has become a popular method for surgical treatment of early stage breast cancer. This technique was described by Toth and Lappert in 1991 [1]. It consists of a standard mastectomy with resection of nipple-areola complex and biopsy scar that preserves the native skin envelope as much as possible. Preservation of inframammary fold and breast contour facilitates immediate breast reconstruction and provides an ideal color and texture match of the reconstructed breast and the opposite breast. The small scar of SSM could be concealed in periareolar location, this and the low probability of nipple-areola complex involvement in early breast cancer [2-5], has made skin sparing mastectomy with nipple- areola complex preservation as an ideal method regarding oncological safety and cosmetic results in selected cases [6].

The risk of skin involvement in T1 and T2 breast carcinoma is very small [7] and the local recurrence after skin sparing mastectomy is a reflection of tumor biology rather than the amount of skin preserved [8-10].

A United King study in 2004 found that 95, 85 and 63 percent of breast surgeons would consider using SSM for DCIS, T1 and T2 tumors respectively, and 17 percent would consider the procedure for the treatment of T3 tumors [11]. Many studies have evaluated the local recurrence rate and survival rate of SSM and immediate reconstruction in early breast cancer [12-15]. The incidence of local recurrence after SSM has been reported as 0 – 7% [16,17].

The purpose of this study has been to evaluate postoperative morbidity, aesthetic result and safety of SSM in the management of early breast cancer in our department.

## Patients and methods

Ninety-five consecutive patients were reviewed in this study that were operated on by the skin-spare mastectomy procedure for their early breast cancer (stage 0, 1, and 2) and followed by immediate breast reconstruction surgery at surgical oncology department, Lyon Sud hospital from April 1995 to April 2003. Chart review was done by one surgeon (J.Y.B). Only the patients were included in this study that were followed for at least four years. Follow-up records gathered from patient's surgical files completed by their surgeon AJCC staging system [18] was utilized to classify breast cancers. Indications for operation were categorized as; primary breast cancer including those with multicentric tumors or the ones with positive surgical margins after second lumpectomy (n = 73, 76.8%); recurrence following breast conservation surgery and adjuvant radiotherapy (n = 20, 21.1%); deformity and microcalcification after breast conservation surgery (n = 1,), and prophylactic mastectomy (n = 1). All the patients were discussed regarding different options for their breast reconstruction surgery before admission.

Skin sparing mastectomy has been classified according to the type of surgical incision, the amount of skin to be removed, and the pattern of skin removal. The choice of incision was chosen according to the size of breast, the location of the previous biopsy scar, location of the tumor and to the surgeon preference. Tennis Racquet and periareolar incisions comprised the most common type of the incisions in our series.

There was Periareolar incisions when a core needle biopsy was done or a prophylactic mastectomy was planned. A sentinel node biopsy or an axillary dissection was performed as indicated. Obviously, patients with positive sentinel node underwent axillary lymph node dissection.

Immediate breast reconstruction techniques were either autologus tissue transfer muscle flaps (*Latissimus dorsi *or *Rectus abdominis*) or implants. Nipple- areola complex reconstruction were planned to be done three months afterward as a separate procedure. A mamoreduction procedure for the opposite breast was performed in the first reconstruction operation session.

Adjuvant chemotherapy and radiotherapy were scheduled when indicated according to the tumor characteristics and stage of the disease. There were no delay in adjuvant therapies in case of any given breast reconstruction complications.

Follow up protocols included a 3- or 6-month clinical review and annual mammography. Patients' median follow up was 69 months (48 to 144). Patients were followed until April 2007 in this study. All data were entered into a dedicated data base (Microsoft Access 2000) and were analyzed using SPSS 11.5 for windows.

## Results

The mean age of patients was 51.6(range 33–72) and most of them (n = 82, 86.3%) were perimenopause and postmenopause women who were referred because of abnormality in screening mammography (microcalcification in 76 (80%) patients, nodule in 2 (2%) patients, other abnormalities in 4 (4%) patients). Only 13 (13.7%) patients were symptomatic (seven (7.4%) patients with mass, six (6.3%) patients with discharge and pain).

Positive family history was recorded in 24 (25.3%) patients (first degree in 18 (18.9%) and second degree in six (6.3%) patients). The diagnosis of breast cancer was histologically proven by core cut or needle biopsy in 34 (35.8%) patients or by open biopsy in 61 (64.2%) patients.

The American Joint Cancer Congress staging were 0(n = 51, 53.7%), I (n = 20, 21.1%), II (n = 2, 2.1%), recurrent (n = 20, 21.1%)(table 1). There was one case of atypical lobular hyperplasia with the history of invasive lobular cancer in opposite breast and one case of deformation and microcalcification after breast conservative therapy.

**Table 1:** Tumor Characteristics

**Variable**	**No of patients (%)**
***Tumor classification***	
Non invasive	58 (61%)
Comedo	36(37.8%)
non-comedo	22(23.1%)
Invasive	35 (36.8%)
***Tumor location***	
Central	13 (13.6%)
Upper pole	37 (38.9%)
Lower pole	22 (23.1%)
Multicentric	22 (23.1%)
***AJCC Staging***	
0	51 (53.6%)
1	20 (21%)
2	2 (2.1%)
Recurrent	20 (21%)
***Grading of the invasive tumors ***	
I	8 (8.4%)
II	20 (21%)
III	7 (7.3%)

In the first half of study (1995–1999), most of the operations were performed by tennis racquet incision and in the second half (1999–2003) most of them were performed by periareolar incision. This shift may be due to more early diagnosis of breast cancer and more use of stereotactic technique in diagnosis of breast carcinoma. All the margins of mastectomy were negative and the rate of malignant involvement of nipple-areole complex was 6.3%. Management of axilla includes: sentinel node biopsy (n = 13, 13.6%), axillary dissection (n = 24, 25.2%) with 11 (11.5%) sampling, 13 (13.6%) conventional dissection, and with no intervention (n = 45, 47.3%). Thirteen patients had history of previous axillary dissection at the time of breast conservative surgery. Sentinel nodes were positive only in two patients who underwent subsequent axillary dissection.

For immediate breast reconstruction, we preferred the *Latissimus dorsi *flap (n = 63, 67%) completely mobilized by dividing of humeral head. A permanent implant was inserted under flap in 28 (29%) patients to achieve optimal volume. TRAM flap was used only in 4 (4%) patients who were obese and required a voluminous flap. Implant reconstruction was used only when the patient (n = 28, 29%) did not accept any additional incision on the skin.

Surgical complications are recorded in table 2 separately according to the type of reconstruction. The most common complication in *latissimus dorsi *group was seroma formation in donor site (n = 20, 31.8%) which was managed most often conservatively, open drainage was needed in 3(15%) patients.

**Table 2:** Complications of Skin Sparing Mastectomy and immediate reconstruction according to the type of reconstruction

**Complication**	**LD ± P**	**TRAM**	**Prosthesis**
**Flap related**			
Partial skin envelope necrosis	3 (4.8%)	0	3 (10.8%)
Partial Flap necrosis	1 (1.6%)	0	0
Periareolar dehiscence	2 (3.2%)	0	0
Contracture and need denervation	1(1.6%)	0	0
**Donor site related**			
Seroma	20(31.8%)	1 (25%)	2(7.1%)
Haematoma	4 (6.3%)	0	0
Superinfection of seroma/haematoma	6 (9.5%)	0	0
Back pain	8 (12.7%)	0	0
Hernia	0	1(25%)	0
**Implant related**			
Displacement	0	0	7 (25%)
Capsular formation	0	0	2 (7.1%)

**Total**	**45(71.4%)**	**2(50%)**	**14(50%)**

Skin loss in breast envelope flap requiring debridement and local wound care occurred in 6 (6.3%) patients, four (66.6%) underwent resection and primary closure (including three implant removals) and two (33.3%) healed by secondary closure. Three of them (50%) had history of breast radiation and nobody was smoker.

Hospital stay was 7.7 days (range 3–19). Eighteen Patients (18.9%) received adjuvant systemic chemotherapy. Adjuvant Tamoxifen was given to 31(32.6%) patients. Postoperative radiotherapy was given to 3 (3.2%) patients.

Contra-lateral surgeries including reduction mammoplasty and mastopexy were done in 18 (18.9%) patients at the same time of nipple-areole reconstruction. Minority of patients in this study (n = 11, 11.5%) have needed implant exchange either because of deformation, displacement or achieving a more symmetry. Contra-lateral surgery was needed in 18(18.9%) patients, confirming better symmetry and decreasing the rate of contra-lateral surgery after skin sparing mastectomy in comparison with non-skin sparing mastectomy.

The final aesthetic results were recorded by another surgeon (M.E) visiting the patients in clinic at least 6 month after operation. There were classified as excellent (n = 34, 35.8%), good (n = 54, 56.8%), and fair (n = 7, 7.3%) according to the Lowery Scaling System [19].

There was one case of regional recurrence in axilla 41 months after skin sparing mastectomy for an in situ carcinoma. There was no invasive component in the mastectomy specimen of this patient. With more evaluation of this case distant metastasis were found in the bone and liver and the patient died in 10 months despite systemic therapy. There was another case of distant metastasis in liver 26 months after treatment of an invasive node positive carcinoma; the patient died in 8 months after diagnosis of distant metastasis.

There was one case of second primary invasive cancer of the opposite breast after two years elapsed of the primary cancer, which was treated by the same SSM technique. One smoker patient developed metachronous metastatic lung cancer five years after treatment for her breast carcinoma. The patient died during last follow up. Three patients died because of cardiac events.

## Discussion

Rising popularity of skin sparing mastectomy is due to better understanding of tumor biology and pattern of recurrence. Data showed that most of patients with local recurrence would progress to distant metastasis and the local recurrence could not be considered as an isolated event resulting from inadequate resections. As with all other types of mastectomy, SSM leaves some residual breast tissue behind but it has been proved that the stage of the primary tumor is the dominant predictor of local recurrence rather than the amount of tissue remains under skin flap.

In SSM the endangered breast tissue could be removed with safe margins while the spared skin could still function cosmetically. The ideal SSM would have flap thin enough to remove all breast tissue, but thick enough to support an adequate blood supply. Torresan *et al*. [20], showed a high prevalence of glandular breast tissue and residual disease in the skin flap thicker than 5 mm. As with standard mastectomy, obtaining free surgical margins is essential to skin sparing mastectomy.

The inframammary fold could be left undisturbed and the thickness of the flap could be the same as those in modified radical mastectomy. Carlson *et al*. [21] examined the inframammary fold tissue in patients undergoing skin-sparing mastectomy. They found breast tissue in 13 out of 24 specimens, but these tissues comprised only 0.02% of the total area. Slavin *et al*. [12] examined 114 skin biopsies from 32 patients undergoing skin-sparing mastectomy, and they found none of the biopsies containing remnant of breast ductal tissue in the dermis. Using SSM, the reconstructive surgery has changed from a prolonged procedure to a more rapid operation in which the reconstructive tissue fills the native skin envelope.

While skin flap necrosis is a recognized complication of SSM because the skin envelope's blood supply can become compromised during dissection, this could be avoided by selecting patients appropriate for the procedure. Nicotine, previous radiotherapy, diabetes and obesity increase the risk of skin envelope ischemia, skin necrosis and infection. These factors could amplify these complications additively, so they should be fully explained to patients before obtaining consent for the operation [22]. Skin flap necrosis has been estimated to occur in 11% of SSM as well as non-SSM cases [13]. In this study we observed very low level of morbidity associated with this procedure. There were six patients (6.3%) with skin envelope ischemia in our series, and three of them (50%) with the history of breast irradiation.

Adjuvant treatment does not seem to be commonly delayed for a possible skin necrosis following SSM and immediate breast reconstruction [23,24], although extensive skin envelope necrosis could delay adjuvant treatment in a few individuals affected.

Having done SSM, the overall survival and the local recurrence rate has been reported to be similar to the cases underwent modified radical mastectomy [1,12,14,25]. In this retrospective study we didn't compare the rate of recurrence between skin sparing and standard mastectomy because we had selected the best-prognosis patients with very small tumor for skin-sparing mastectomy and this selection bias would affect any conclusion.

Although the follow up time in our series has not been long enough, there were few studies in which the follow up time of SSM reach as long as 6 years [9,16,25,26]. In the study reported by Spiegel *et al*. [26], the follow up time after SSM is at least six years and the incidence of local recurrence was 5.5% for invasive carcinoma and 0% for *in situ *carcinoma. In the current study we had only one recurrence of tumor in the axilla of a patient with ductal carcinoma in situ 41 months after operation This low rate of local recurrence is similar to prior published series (table 3).

**Table 3:** local recurrence rate after Skin Sparing Mastectomy and immediate reconstruction in early breast cancer in previously published papers

Author	Number of cases	Median follow-up (months)	Local recurrence (%)
Gerber [2]	112	59	5.4
Carlson [13]	327	42	4.8
Carlson [32]	565	64.6	5.5
Carlson [34]	223	82.3	4
Slavin [12]	51	45	2.0
Kroll [15]	104	>60	6.7
Kroll [16]	114	72	7
Fersis [17]	60	52	6.6
Rivadeneira [25]	71	49	5.6
Medina-Franco [9]	173	73	4.5
Spiegel [26]	177	72	5.6
Newman [29]	437	50	6.2
Toth [30]	50	57	0
Singletary [31]	545	<60	2.6
Peyser [23]	71	24	3
Greenway [33]	225	49	1.7
Current study	95	59	1.1

We propose that the low rate of local and systemic failure in our series could be as result of our studied population, which has been a special subset of very early breast cancer with inherently good outcome. Ubiruba *et al*. [27], reported three cases of local recurrence at the needle biopsy site in patients treated with SSM whose diagnoses were obtained through sterotactic needle biopsy. Thirty-six percent of our patients had history of core needle biopsy but fortunately without any local recurrence at the needle biopsy site.

Although the majority of our patients had in situ breast cancer and small invasive breast carcinoma with extensive in situ component, more recently SSM has been used to treat more advanced disease with local recurrence rates increasing with more advanced stages [28].

## Conclusion

In conclusion SSM appears to be oncologically safe for early breast cancer (stage 0-II), but its use for more advanced stages require more prospective analysis.

## Competing interests

The authors declare that they have no competing interests.

## Authors' contributions

RO carried out data collection and drafted the manuscript. JYB carried out all the surgical procedure and followed the patients. ME carried out aesthetic evaluation and participated in drafting the manuscript. All authors read and approved the final manuscript.
